# Polymorph Separation by Ordered Patterning

**DOI:** 10.3390/molecules27217235

**Published:** 2022-10-25

**Authors:** Massimiliano Cavallini, Marco Brucale, Denis Gentili, Fabiola Liscio, Lucia Maini, Laura Favaretto, Ilse Manet, Massimo Zambianchi, Manuela Melucci

**Affiliations:** 1 Istituto per lo Studio dei Materiali Nanostrutturati, CNR- Via P. Gobetti 101, 40129 Bologna, Italy; 2 Istituto per la Microelettronica e Microsistemi, CNR, Via P. Gobetti 101, 40129 Bologna, Italy; 3 Deptartment of Chemistry ‘‘G. Ciamician’’, Università di Bologna, Via F. Selmi 2, 40126 Bologna, Italy; 4 Istituto per la Sintesi Organica e la Fotoreattività, CNR, Via P. Gobetti 101, 40129 Bologna, Italy

**Keywords:** polymorphism, patterning, polymorph control

## Abstract

We herein address the problem of polymorph selection by introducing a general and straightforward concept based on their ordering. We demonstrated the concept by the ordered patterning of four compounds capable of forming different polymorphs when deposited on technologically relevant surfaces. Our approach exploits the fact that, when the growth of a crystalline material is confined within sufficiently small cavities, only one of the possible polymorphs is generated. We verify our method by utilizing several model compounds to fabricate micrometric “logic patterns” in which each of the printed pixels is easily identifiable as comprising only one polymorph and can be individually accessed for further operations.

## 1. Introduction

In material science, polymorphism is defined as the ability of a single compound to assume two or more stable crystalline forms [1,2,3,4]. It is a widespread phenomenon transversal to all scientific disciplines involving solid materials.

As the crystalline structure has a fundamental role in determining the chemical and physical properties of a given material, distinct polymorphs can dramatically differ in their properties [5,6,7,8,9,10,11]. This effect severely impacts the technological application of materials. Practical examples range from semi/superconductors to food and explosives [7,12,13]. Usually, technological applications require the use of a specific polymorph [14]. In this respect, in recent years, an impressive effort has been devoted to driving polymorph formation/selection toward controlled, specific structures by acting on material processing [15,16], patterning [17], and postdeposition procedures, such as thermal and vapor solvent treatments [18,19] or mechanical processing [20].

On the other hand, polymorphism can itself be an added value [4] as it originates new functionalities or tunes existing ones [21,22]; moreover, the scouting of different crystal forms and the study of the specific properties of selected polymorphs can be an efficient way to select the best performances among the possible structures and to prevent the growth of undesired crystalline forms. In addition, the exploration of several polymorphs for a new molecule is often the way to best exploit it, leading also to the finding of new, unexpected applications [23,24]. Due to this, it is mandatory to quickly and cheaply screen the different possible polymorphs of a given compound while allowing for their individual characterization in isolation.

Here, we address the problem of polymorph separation by introducing an original procedure based on surface patterning aimed at separating polymorphs in a controlled manner and storing them on an addressable structure. 

In our method, isolated polymorphs are patterned within individually accessible micrometric structures, each containing only a single polymorph. With this aim, we fabricated a micrometric logic pattern (i.e., a pattern in which each pixel is precisely addressable) in which each printed pixel comprises only a single polymorph and can thus be individually accessed for further operations.

**Figure 1:**
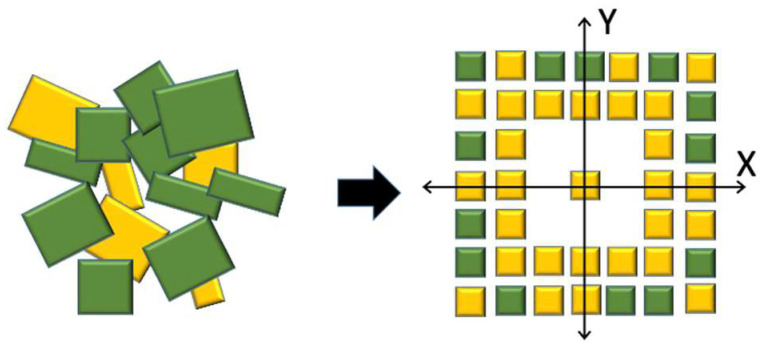
Concept of patterning approach for polymorph separation: Left, polymorph agglomerates; Right, separated addressable structures made by a single polymorph. The geometry reproduces an Aztec code in which the central point is the finder pattern of the code.

Our approach was inspired by the simple observation that a complex system formed by different objects (in our case, the agglomeration of crystals of polymorphs) can be simplified by its ordered spatial separation based on the smallest homogeneous unit (in our case, the stable nuclei of a specific polymorph). Figure 1 shows the concept behind our approach. 

An ad-hoc patterning strategy based on appropriately small individual units (i.e., the smallest crystallite containing a unique polymorph) enables the facile and fast separation of each polymorph. Importantly, our approach is independent of the nature of the polymorphs formed inside each printed structures, as it allows the recognition of the nature of polymorphs by their fluorescence emission and their precise position according to their spatial coordinates with respect to reference points (finder pattern) in the surface. 

As a proof of concept, we applied this strategy to thin deposits of polymorphs, using the smallest observable crystallite formed by a single polymorph as the basic building block. The basic building block was fabricated exploiting the fact that growing a polymorph from solution in appropriate confinement (i.e., inside a box having a sufficiently small size), only one type of polymorph can be formed inside each box [25]. This behavior is well-known [24] and occurs when the accretion of monomeric material to the growing crystal is sufficiently faster than the formation rate of new crystallization nuclei, such that each growing crystallite reaches the size of the whole box before the formation of a second stable nucleus in the same box. In addition, this effect is enhanced by the secondary nucleation effect, i.e., the first-formed crystal locally favors the formation of other crystals with the same structure [26,27], which further contributes to the formation of a single type of polymorph in each box. The behavior depends on the specific compound and, despite some possible effects exerted by confinement on the crystal growth, can be controlled acting on the concentration of the solution, thus controlling when the solution reaches supersaturation inside the box or, as in our case, acting on the size of confinement [28]. The box-size can, in principle, range from sizes comparable with the crystallization nuclei (i.e., a few nanometers) to the macroscopic. In our method, the boxes are provided by a printing method capable of forming polymorphs in confined conditions. Noticeably, the printing process can influence the formation of polymorphs in several ways; in particular, the deposition in confinement that must be used to pattern the crystals normally promotes the formation of crystal domains which are larger than those formed via conventional thin-film growth methods, such as drop-casting or spin-coating, and can, in some cases, induce crystal orientation [28,29]. This behavior can be ascribed to the reduced evaporation rate of solvents and to the limited molecular diffusion inside the box during the crystallization [28]. This contribution is helpful to our aim because it generally enhances the selection effect of the patterning and the crystalline domain size of printed structures, as compared to that observed for film prepared by conventional methods. Eventually, confinement can drive the polymorph formation toward a specific polymorph [30,31,32], which could be different from the stable species formed by conventional deposition [33]. Other parameters that can play an active role toward the formation of a specific polymorph can be locally imposed by the nature of the surface [34], stamp [29], solvent, or by the shrinkage rate [24] and temperature [31].

**Figure 2:**
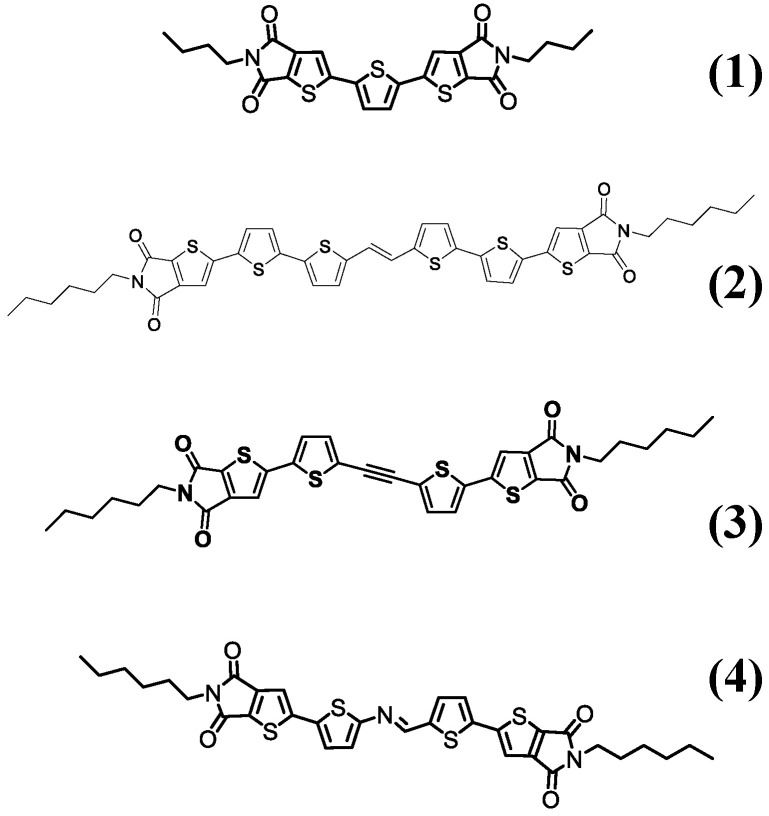
Chemical structures of compounds (**1**) 2,2′(thiophene-2,5-diyl)bis(5-butyl-5Hthieno[3,2-c]pyrrole-4,6-dione); (**2**) (E)-2,2′-(5′,5″‘-(ethene-1,2-diyl)bis([2,2′-bithiophene]-5′,5-diyl))bis(5-hexyl-4H-thieno[2.3-c]pyrrole-4.6(5H)-dione); (**3**) 2,2′-(5,5′-(Ethyne-1,2-diyl)bis(thiophene-5,2-diyl))bis(5-hexyl-4H-thieno[2,3-c]pyrrole-4,6(5H)-dione); and (**4**) (E)-5-Hexyl-2-(5-(((5-(5-hexyl-4,6-dioxo-5,6-dihydro-4H-thieno[2,3-c]pyrrol-2-yl)thiophen-2-yl) imino)methyl)thiophen-2-yl)-4H-thieno[2,3-c]pyrrole-4,6(5H)-dione.

To demonstrate polymorph separation by patterning, we used several known compounds capable of forming stable and easily recognizable polymorph structures of a micrometric size (and thus optically accessible and easily available for further operations). In particular, we used 2,2′(thiophene-2,5-diyl)bis(5-butyl-5Hthieno[3,2-c]pyrrole-4,6-dione) (herein designated as compound **1**) [24] as the model material; (E)-2,2′-(5′,5″‘-(ethene-1,2-diyl)bis([2,2′-bithiophene]-5′,5-diyl))bis(5-hexyl-4H-thieno[2.3-c]pyrrole-4.6(5H)-dione) (herein designated as compound **2**) [35]; 2,2′-(5,5′-(Ethyne-1,2-diyl)bis(thiophene-5,2-diyl))bis(5-hexyl-4H-thieno[2,3-c]pyrrole-4,6(5H)-dione) (herein designated as compound **3**) [35]; (E)-5-Hexyl-2-(5-(((5-(5-hexyl-4,6-dioxo-5,6-dihydro-4H-thieno[2,3-c]pyrrol-2-yl)thiophen-2-yl) imino)methyl)thiophen-2-yl)-4H-thieno[2,3-c]pyrrole-4,6(5H)-dione (herein designated as compound **4**) [35]; the chemical structures of which are shown in Figure 2. All of these compounds form different polymorphs of a micrometric size that are well-distinguishable via their different fluorescence emission spectra.

## 2. Results and Discussion

**Figure 3:**
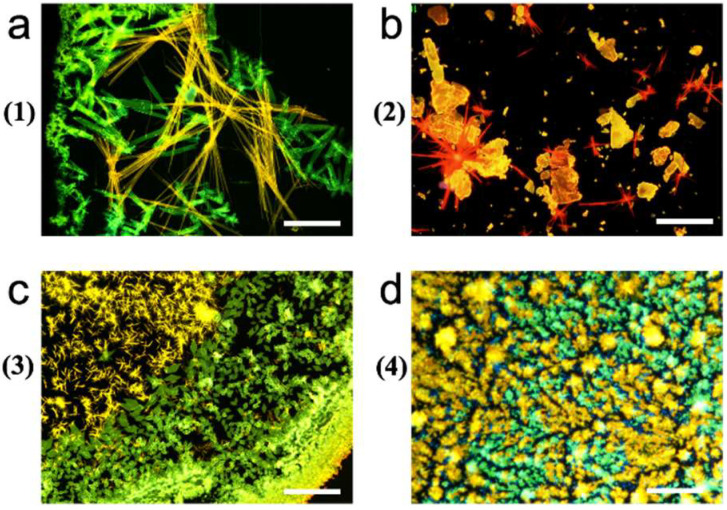
Fluorescence images of polymorphic, thin deposits of (**a**) Thieno(bis)imide end-functionalized terthiophene (**1**); (**b**) 2,3-thieno(bis)imide-ended oligothiophenes bearing unsaturated ethylene (**2**); (**c**) 2, 3-thieno(bis)imide-ended oligothiophenes bearing inner bridges (**3**); and (**d**) 2,2′-(5,5′-(Ethyne-1,2-diyl)bis(thiophene-5,2-diyl))bis(5-hexyl-4H-thieno[2,3-c]pyrrole-4,6(5H)-dione) (**4**).

When deposited on a variety of technologically relevant substrates (glass, silicon, and gold) compounds **1**, **2**, **3**, and **4** form two types of concomitant polymorphs (i.e., different polymorph domains that form under identical conditions), each exhibiting a distinct fluorescence emission [24]. The mean size of crystals can be controlled by the shrinkage rate (i.e., by drop-casting in air or in an atmosphere saturated with solvent vapor) from a few microns to a few hundred microns; moreover, the compounds can be easily processed in optically accessible micrometric structures by a variety of wet lithographic methods [19,24,36]. Figure 3 shows representative fluorescence images of polymorphic, thin deposits of compounds **1**, **2**, **3** and **4**.

Printing was performed via lithographically controlled wetting (LCW), which is a simple and versatile method largely used to pattern functional materials and to grow micro- and nano-crystals in confinement with size and positional control [28,29]. Although we chose LCW for its convenience, the procedure is not related to the patterning method, and similar results were obtained by patterning the same model compound by microtransfer molding or spatially controlled demixing [36].

In order to evaluate the critical pixel size needed to obtain only one polymorph for each printed structure, we investigated the effect of the size of the stamp features on polymorph assembly by printing a 0.5 g/L solution of compound **1** in toluene (see Section 3).

**Figure 4:**
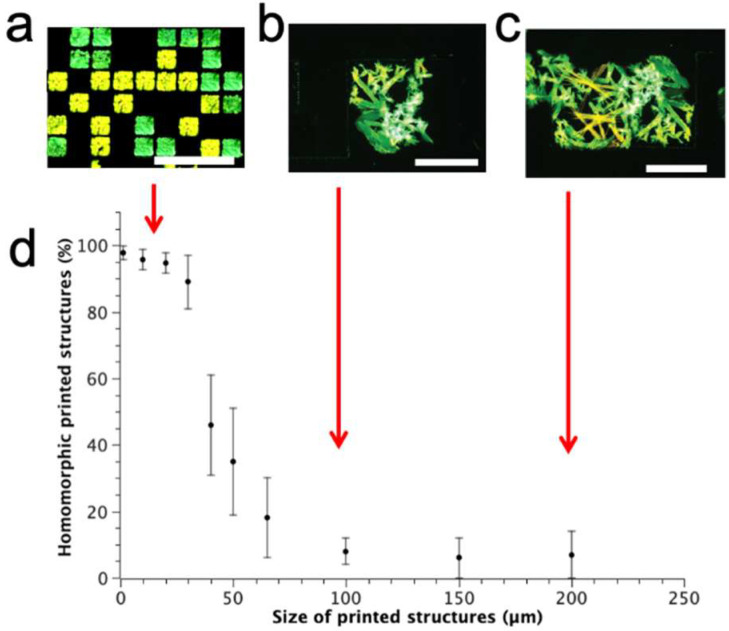
Polymorph composition homogeneity versus confinement size in printed structures of compound **1**: (**a**) Fluorescence image of compound **1** deposited in 20 × 20 µm^2^ structures, (**b**) 100 × 100 µm^2^, and (**c**) 300 × 200 µm^2^. Bars are 80 µm in length; (**d**) Trend of percentage of printed structures containing only one polymorph vs. size.

No confinement effect was observed when using a stamp with pixel sizes above 100 × 100 µm^2^. In this case, the polymorph size and distribution are almost identical to those of thin deposits prepared by drop-casting (Figure 4c); each printed structure contains both of the polymorphic forms with the same percentages observed in the drop-cast film [20]. 

When the box-size started to be comparable with the size of the crystals (<100 × 100 µm^2^), the polymorphs were no longer distinguishable by shape. Scaling-down to a box-size of 50 × 50 µm^2^, more than 30% is formed by only one polymorph. (Note: at this size, the system is particularly sensitive to the experimental condition, and the percentage of polymorph could be variable). Eventually, when patterning structures of 20 × 20 µm^2^ or smaller, more than 98% of them are formed by only one polymorph, thus separating the two polymorphs in an ordered pattern made of homomorphic structures. As an example, Figure 4 shows the polymorphs’ composition versus their size in the printed structures of compound **1**.

**Figure 5:**
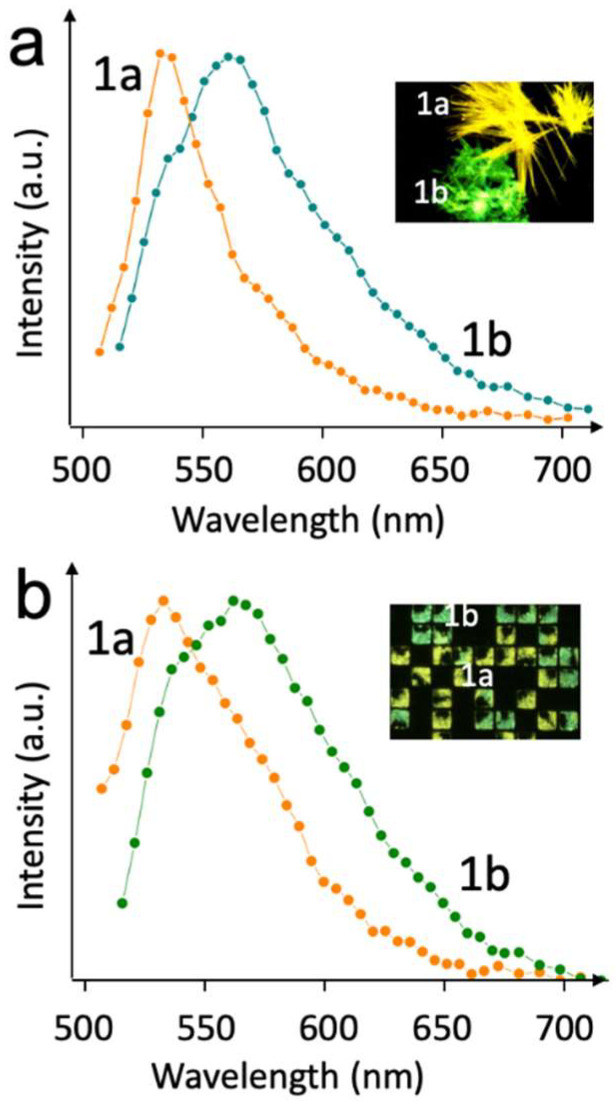
Confocal florescence spectra of (**a**) drop-cast film from toluene solution on silicon; (**b**) Patterned structures of a 20 × 20 µm^2^ size. 1a and 1b refer to the different polymorphs of compound **1**.

Despite some irregularities present in the printed structures, the size and the shape of the crystals are mainly imposed by the features used for printing, making the different polymorphs indistinguishable by shape. However, the different polymorphs are still well-distinguishable by fluorescence, as shown in the images in Figure 4. No evident differences in the fluorescence and photoluminescence spectra were observed when comparing polymorphs printed in confinement with polymorphs grown by drop-casting in the absence of confinement (Figure 5).

**Figure 6:**
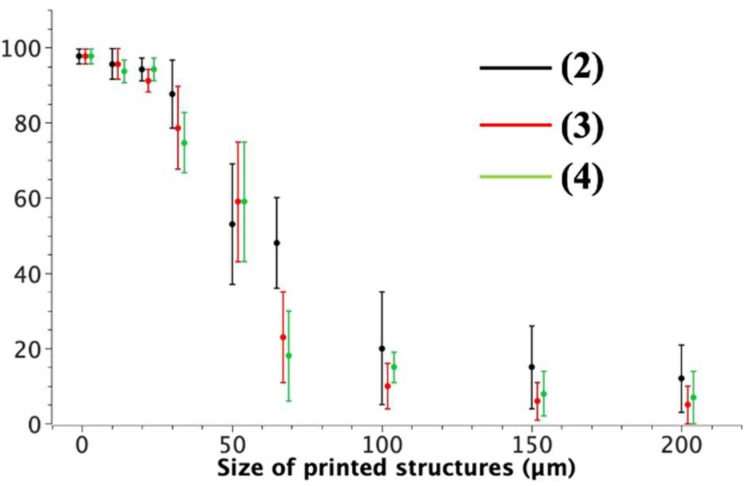
Polymorphs’ composition versus size in printed structures of compounds **2**, **3**, and **4**.

Noticeably, the total percentage of the two polymorphs, obtained by counting the printed structures, is almost the same as that obtained in the in drop-cast film. Moreover, in the rare cases of printed structures containing two polymorphs (<2% for box-size < 20 × 20 µm^2^), they are always formed by two crystals physically separated by a few microns. This occurrence is well-known in LCW, and it is due to the formation of multiple menisci during the printing process [29]. To confirm the generality of the patterning approach to separate polymorphs, we investigated the effect of printing size versus polymorph composition in compounds **2**, **3**, and **4**. All of these compounds are able to form micrometric-sized fluorescent polymorphs similar to those of compound **1**. Figure 6 shows the polymorphs’ composition versus size in printed structures of these compounds.

**Figure 7:**
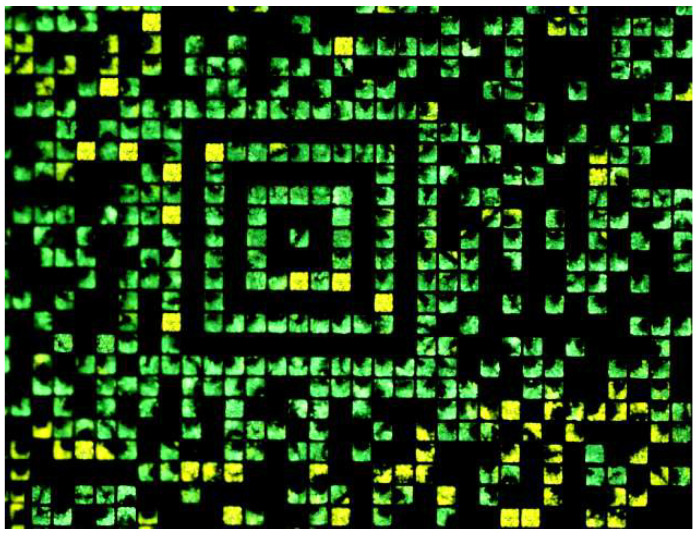
Ordering of polymorphs. Fluorescence image of compound **1** patterned on silicon surface as an Aztec code. Each printed structure is 20 × 20 µm^2^ and contains only a polymorph (green or yellow) in >98% of pixels.

To form accessible, addressable structures made of a single polymorph, compound **1** was patterned, reproducing a 2D barcode made of 20 × 20 µm^2^ square pixels. In particular, we printed an Aztec code, which is a logic pattern used to store information in a 2D barcode. Figure 7 shows a fluorescence image of a pattern printed on a silicon wafer and the PL spectra recorded in different printed structures.

In the printed codes, each homo-polymorphic structure is easily addressable manually and automatically via publicly available software, and the fluorescence color identifies the type of polymorph.

## 3. Materials and Methods

### 3.1. Materials

Compounds **1**–**4** were synthesized according to references in the text.

### 3.2. Lithographically Controlled Wetting

In LCW, a stamp made of PDMS, the motif of which consists of a distribution of a square with a size ranging from 20 × 20 µm^2^ to 300 × 200 µm^2^, is placed in contact with a 20 µL liquid film of solution of **1** (0.5 g/L) on a substrate. The menisci form under the stamp protrusions due to the onset of capillary forces. As the solvent evaporates, the solution remains pinned only to the protrusions, making the region in between the protrusions free of solution. As the solution reaches supersaturation, crystals form in the box formed between the stamp protrusions and the surface. Confinement is imposed by the size of the stamp features. A detailed description of the process and the apparatus used for LCW is reported in [29].

In order to fabricate addressable structures, an Aztec 2D code made of 20 × 20 µm^2^ is used. The substrates consist of a 10 × 10 mm^2^ piece of silicon covered by 200 nm of thermal oxides or glass. It is cleaned by sonication for 2 min. in electronic-grade water (milli-pure quality), 2 min. in acetone, and then 2 min. in 2-propanol. Thin deposits of **1** are prepared by drop-casting 20 µL/cm^2^ of a 1 g/L solution in toluene or chloroform on Si/SiO_2_ wafers. The solvent is slowly evaporated at room temperature in a solvent-saturated atmosphere in the same substrates used for patterning. 

### 3.3. Fluorescence Microscopy

Fluorescence images were recorded with a Nikon i-80 microscope equipped with epifluorescence (FM) using FM filters Nikon Ex 420, DM 435, BA 475, Ex 535, DM 570, and BA 590. The FM images were recorded using a commercial CCD camera (Nikon CCD DS-2Mv). The illumination was performed by a 100 W halogen lamp at fixed power (i.e., tension 12 V) and with a fixed time of acquisition of the CCD (500 ms). 

### 3.4. Laser Scanning Confocal Fluorescence Microscopy

Fluorescence spectra were obtained by laser scanning confocal fluorescence microscopy. It was performed on an inverted Nikon Ti-E microscope (Nikon Co., Shinjuku, Japan) equipped with a 405 nm pulsed/CW diode laser (PicoQuant GmbH, Berlin, Germany) and a Nikon A1 spectral detector module consisting of a multi-anode photomultiplier with an array of 32 anodes. A wavelength bandwidth of 6 or 10 nm per anode was applied.

## 4. Conclusions

In conclusion, we proposed a straightforward concept for the ordering of polymorphs by patterning, which allows the separation of polymorphic crystals into ordered and accessible structures. We used standard, established methods and model materials as a proof of concept, but the approach is general and can be extended to other soluble materials that are able to form different polymorphs, as well as to any patterning method. 

Here, we used fluorescent materials for the simplicity of identification; however, the method was successfully applied, exploiting other properties related to polymorphism, such as the change of colors using spin crossovers [16] and birefringence in azobenzene [37] compounds.

Independent of the printing method, ordered patterning has no formal limitation since the key parameter is the box-size, which is not related to the chemistry or the crystallization process of the system, and can thus, in principle, be applied to any compound processable by wet methods. This work can suggest new routes for controlling polymorph crystallization toward the full exploitation of the functional properties of many materials. In forthcoming works, we will extend the process by the local guiding of the nature of each polymorph, acting on the chemical differentiation of the stamp motif, enlarging the prospective of the proposed approach toward the qualitative and quantitative control of polymorphism.

## Data Availability

Experimental data are available from the corresponding author.

## Sample Availability

Samples of the compounds are not available from the authors.
